# Brd4 inhibition ameliorates Pyocyanin-mediated macrophage dysfunction via transcriptional repression of reactive oxygen and nitrogen free radical pathways

**DOI:** 10.1038/s41419-020-2672-0

**Published:** 2020-06-15

**Authors:** Feimei Zhu, Feng Xiong, Jinchen He, Keyun Liu, Yuanyuan You, Qian Xu, Junming Miao, Yu Du, Lijuan Zhang, Hongyu Ren, Xiaoying Wang, Junli Chen, Jingyu Li, Shanze Chen, Xiaokang Liu, Ning Huang, Yi Wang

**Affiliations:** 1 Department of Pathophysiology, West China College of Basic medical sciences & Forensic Medicine, Sichuan University, 610041 Chengdu, China; 2 Department of Cardiology, The third People’s Hospital of Chengdu, 610031 Chengdu, China; 3 Department of Physiology, School of Medicine, Hubei University for Nationalities, 445000 Enshi, China; 4 Department of Pharmacology, West China College of Basic medical sciences & Forensic Medicine, Sichuan University, 610041 Chengdu, China

**Keywords:** Biochemistry, Apoptosis

## Abstract

Macrophages play critical roles in the first-line immune defense against airway infections caused by *Pseudomonas aeruginosa* (PA). The redox-active phenazine-pyocyanin (PCN), as one of the most essential virulence factors, facilities PA-related infection via a wide spectrum of cellular oxidative damages. However, little is known for PCN cytotoxicity in macrophages. In this study, besides showing PCN-mediated reactive oxygen species (ROS) indeed involved in macrophage viability and function impairment, we at the first time demonstrated a novel role of reactive nitrogen species (RNS) pathway causing cellular damage in PCN-challenged macrophages. Using small molecule inhibitor JQ1 targeting Bromodomain and extra-terminal family proteins, we showed restrained iNOS-dependent nitric oxide (NO) production correlated with abolished Brd4 recruitment to the *NOS2* (encoding inducible nitric oxide synthase-iNOS) promoter. Application of JQ1 diminished PCN-mediated peroxynitrite (ONOO^−^) that followed ROS and NO induction, restored macrophage survival and bacteria clearance as well as repressed local inflammation in PA/PCN-challenged mice lungs. Our results uncover a novel link between PCN-mediated macrophage dysfunction and reactive free radicals that rely on Brd4-dependent transcription modulation of multiple stress-response genes, suggesting Brd4 could be a promising therapeutic target in treating PA-related lung infection.

## Introduction

PA is the leading cause of significant morbidity and mortality in hospitalized patients with acute and chronic lung infections, such as cystic fibrosis (CF), non-CF bronchiectasis, and chronic obstructive pulmonary disease (COPD)^1^. The redox-active PCN, which is considered one of the most essential virulence factors is frequently found in patients’ sputa (concentration varies from 0 to 131 μM)^2^ and negatively correlates with lung function^3,4^. It has been reported that PCN triggers neutrophil apoptosis^2^ and disturbs airway epithelial cell function to counteract host innate immune response^4^. Although the exact roles of macrophage against PA infection in lungs are still in debate^5^, results from multiple studies have implicated that alveolar macrophages take parts in the first-line immune response, which could be largely repressed by PCN^6,7^. Therefore, it is important to dissect the detailed mechanisms of PCN-mediated macrophage dysfunction and explore potential detoxification strategy to enhance host innate immune defense during PA infection.

To date, many in vitro studies have revealed that PCN causes cellular disturbance via oxidative stress^8,9^ by generating reactive oxygen species (ROS) and suppressing anti-oxidant pathways^3^. Mis-regulated cellular redox-oxidant status results in oxidized protein adduct formation and various cellular dysfunctions^10^. However, the sufficiency of ROS in carrying out PCN cytotoxicity is questionable as there still lacks evidence for the application of anti-oxidant treatments in lung infections^11^. On the other hand, iNOS-dependent intracellular NO has been shown to link with multiple cellular responses to outer stimulations such as ischemia and Lipopolysaccharides (LPS) stimulation^12,13^. Intracellular NO specifically serves as a bactericidal reagent as well as messenger molecule mediating host immune response in monocytes whereas the abnormality of its balance was reported to cause direct cytotoxicity through ONOO^−^—the product from reactions between NO and ROS (O_2_^•−^)^14^. As the central regulatory factor to maintain redox-oxidative homeostasis, Nuclear factor (erythroid-derived2)-like 2 (Nrf2) and its binding partner Kelch-like ECH-associated protein 1 (Keap1) are shown to restrict intracellular ROS overloaded^15^. Meanwhile, NO production is also under accurate controls of three different nitric oxide synthases (NOS) among which iNOS is in charge of generating inducible NO and subjects to tightly transcriptional regulation by various stressors^10^.

Precise transcriptional controls of key stress-response genes (including ROS and NO pathway genes) require the participation of multiple basal transcription factors. BET family proteins (Brd2, Brd3, Brd4, and testis-specific Brdt proteins) have been found to play fundamental roles in helping transcriptional adaption to encounter various extracellular challenges^16–18^. BET proteins act as readers of acetylated histone lysine residues through two conserved bromodomains (BD1 and BD2) while their extra-terminal and C-terminal domains modulate gene transcription by recruiting transcription activators and histone modifiers to promoters and enhancers, resulting in enhanced transcription activity of RNA polymerase II (PolII)^19^. Small-molecule BET inhibitors, mimicking the histone acetyl-lysine moiety disassociate BET proteins from acetylated chromatin^20^ thereby rendering potent anti-tumor effect by targeting *MYC* transcription while suppressing inflammation and tissue fibrosis via disrupting pro-inflammatory gene expression^17,21^. Among BET family members, Brd4 is particularly known to recognize multiple acetylated lysine sites on histone H3 and H4 (K14 in H3, either H4K5 and K12, or K8 and K16 in H4) to largely facilitate transcription activation by recruiting Positive Transcription Elongation factor complex (P-TEFb), Cyclin dependent kinase-9(CDK9) and mediator complex^22^. It has been found that Brd4 enhances NO synthesis via stimulating iNOS expression in bacteria-infected macrophages^23^ and regulates Nrf2-dependent anti-oxidation pathways^16,24^, suggesting the involvement of Brd4 in controlling oxidative free radical homeostasis during cellular stress. In our study, we found inhibition of Brd4 rescued PCN-induced macrophage death and subsequent function disturbance by suppressing activated ROS as well as iNOS-dependent NO production. JQ1 and shRNA-mediated *BRD4* silencing re-established cellular redox-oxidative balance to get rid of the toxic ONOO^−^ overload, ensuring the proper activation of macrophages upon PCN challenge. Moreover, JQ1 resumed bacterial clearance and harnessed tissue inflammation in PA-infected mice lungs. In summary, our data demonstrated the novel mechanism of PCN cytotoxicity in macrophages via RNS induction that is mediated by transcriptional regulation of Brd4 on ROS and NO pathways, indicating the promising application of JQ1 in supporting host innate immunity during PA-infection.

## Results

### The BET inhibitor (+)JQ1 reduces PCN-mediated macrophage death

**Fig. 1:**
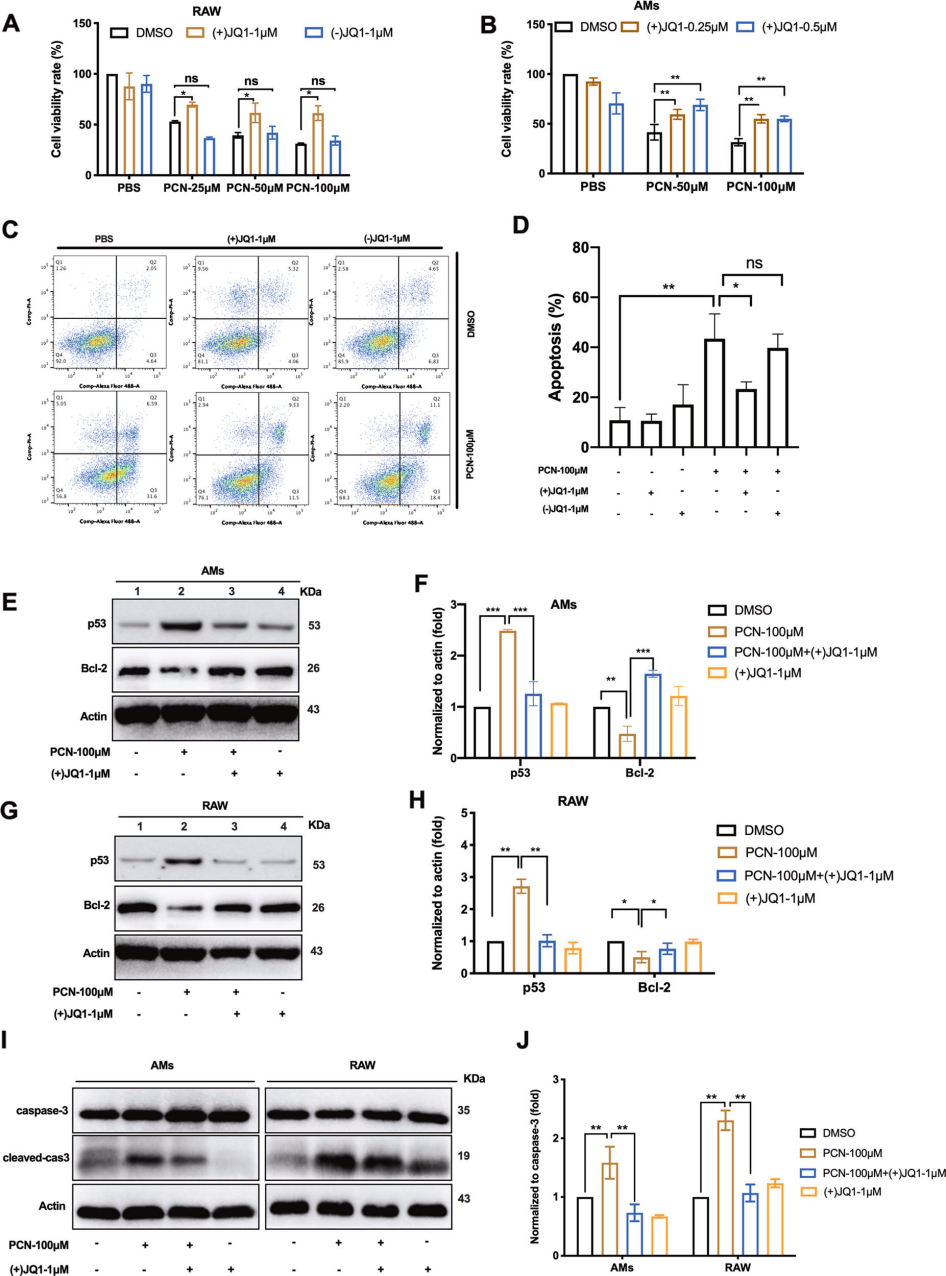
(+)JQ1 reduced PCN-mediated macrophage death. Cells were pre-treated with (+)JQ1 or (−)JQ1 24 h before experiment, unless otherwise specified. CCK8 assay showing the cell viability of **a** RAW and **b** AMs at 24 h post-PCN challenge. **c** Flow cytometry analysis and **d** quantified apoptosis rates of double-stained RAW cells at 8 h post-PCN challenge. AnnexinV-488A (apoptotic fraction: Q2 and Q3 quadrants) and PI (necrotic fraction: Q1 quadrant). Western blot showing the expression of p53 and Bcl-2 in RAW (**g**) and AMs (**e**), respectively, as well as cleaved-caspase3 and total caspase 3 at 8 h post-PCN challenge (**i**). **f**, **h** Densitometry analysis from **e**, **g**. **j** Ratio between cleaved-caspase 3 and total caspase 3 from (**i**). (Data shown were mean ± SD from three independent experiments. *P < 0.05, **P < 0.01compared with indicated group, ns non-significant. One-way ANOVA test followed by *t*-test).

PCN is known to cause cell death in various cell types^2,25^ although its cytotoxic effect in macrophage is unclear. As we expected, 24 h PCN challenge was toxic on RAW264.7 (RAW) cells in a dosage-dependent manner (Fig. 1a black bar). To assess the importance of BET proteins in PCN-mediated macrophage death, BET inhibitor JQ1 ((+)JQ1) and its inactive isomer (−)JQ1 were applied prior to PCN stress. Pre-treatment of 1 μM (+)JQ1 increased RAW cell survival compared with DMSO and (−)JQ1 group respectively (Fig. 1a). Similarly, 0.25 and 0.5 μM (+)JQ1 displayed protective effect on PCN-challenged mice alveolar macrophages (AMs) (Fig. 1b). Moreover, flow cytometry analysis showed that apoptotic death of RAW cells induced by 100 μM PCN (Fig. 1c Lower left compared with upper left) was significantly rescued by 1 μM (+)JQ1 (Fig. 1c lower middle compared with lower left and Fig. 1d). Western blotting analysis confirmed these observations by showing that PCN dramatically increased protein levels of apoptotic factor p53 (Fig. 1e–h) and cleaved caspase 3 (Fig. 1i, j) while decreased Bcl-2 expression (Fig. 1e–h). Consistently, pre-incubation of (+)JQ1 reversed above changes in both RAW and AM cells, suggesting (+)JQ1 significantly rescued macrophage survival from PCN-induced apoptosis. Additionally, we conducted siRNA silencing assay to show substantial reduction of p53 and cleaved caspas-3 expression upon PCN stress (Fig. S1A). Cell viability assays further confirmed the involvement of p53-dependent apoptosis pathway in PCN-induced macrophage death (Fig. S1B–D).

(+)JQ1 regulates oxidant/anti-oxidant genes expression and alleviates PCN-induced ROS production

**Fig. 2:**
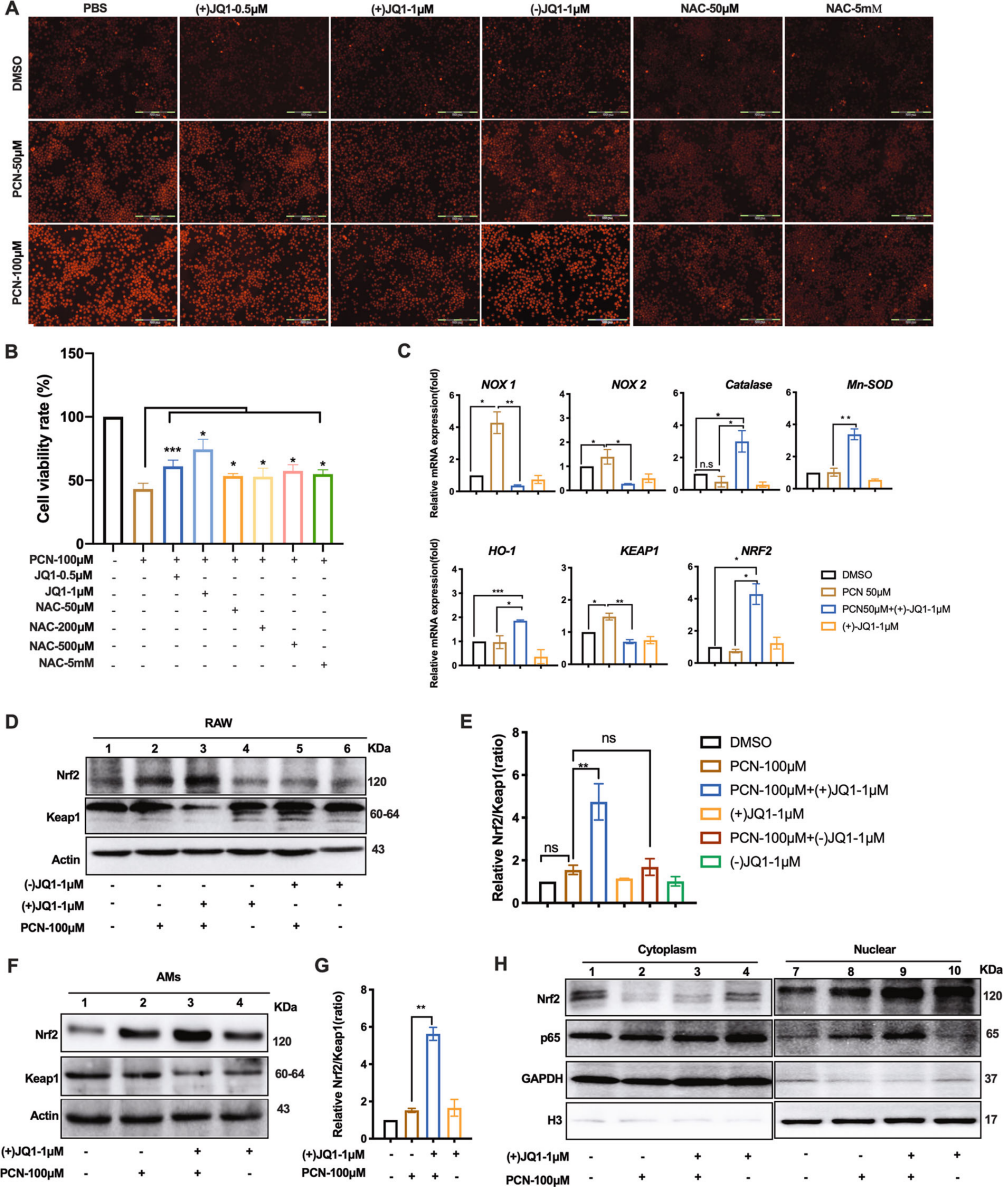
(+)JQ1 regulated oxidant and anti-oxidant pathways in PCN-challenged macrophages. **a** Representative images showing DHE-stained intracellular ROS in RAW cells at 3 h post-PCN challenge. **b** CCK8 assay showing the viability of RAW cells at 24 h post-PCN challenge. **c** RT-qPCR showing the mRNA expression levels of indicated genes in RAW cells at 8 h post-PCN challenge. (*P < 0.05, **P < 0.01 compared with indicated group. Bar graphs represent three independent experiments, each performed in triplicates). **d**, f Western blot showing the expression of Nrf2 and Keap1 in RAW cells and AMs at 8 h post-PCN challenge. **e**, **g** Densitometry analysis of expression ratio between Nrf2 and Keap1 in **d**, **f**. **h** Western blot showing the cytosolic and nuclear distribution of Nrf2 and p65 in RAW cells. (Data shown were the mean ± SD from three independent experiments. *P < 0.05; **P < 0.01 compared with indicated group. One-way ANOVA test followed by *t*-test).

PCN is well-studied to cause neutrophil and epithelium death through elevated intracellular ROS^4,26^. We firstly investigated whether the similar mechanism functions in macrophages. DHE labeling assay was conducted in PCN-treated RAW cells and showed 1 μM (+)JQ1 substantially inhibited intracellular ROS formation (Fig. 2a) while the similar results were also observed in AMs (Fig. S2A). The comparable repression of ROS production (Fig. 2a) and moderate rescuing of macrophage viability were obtained by 50–50 mM ROS scavenger N-acetyl cysteine (NAC) (Fig. 2b). As we further investigated how (+)JQ1 takes part in repressing PCN-mediated ROS production, RT-qPCR data showed induced expression of NADPH oxidase genes, *NOX1* and *NOX2*, were blocked while expression of anti-oxidant genes encoding Catalase, HO-1 and Mn-SOD were enhanced by (+)JQ1 in PCN-challenged RAW cells (Fig. 2c), indicating the role of (+)JQ1 in transcriptionally balancing oxidation and anti-oxidation processes. Noteworthily, epidermal growth factor receptor (EGFR) functioning at the downstream of activated ROS during PCN-caused airway epithelial cell dysfunction^27^　was found to be (+)JQ1-responsive (Fig. S2B) but not involved in PCN macrophage death as its inhibitor (AG-1478) did not interfere with RAW cell survival (Fig. S2C). Moreover, we sought to investigate the expression of Nrf2 and its negative regulator Keap1 due to their guardian roles on ROS homeostasis^28^. RT-qPCR results showed (+)JQ1 treatment up-regulated *NRF2* expression particularly in PCN-stressed cells where substantial reduction in *KEAP1* mRNA was observed (Fig. 2c). Western blotting analysis was consistent with this data in both PCN-treated RAW cells and AMs (Fig. 2d, f). Furthermore, the enlarged ratio between these two proteins in cytoplasm (Fig. 2e, g) and the enhanced nucleus translocation of Nrf2 (Fig. 2h) highlighted the stimulatory effect of (+)JQ1 on Nrf2 signaling upon PCN stress. Interestingly, NF-κB subunit p65 was also found to translocate to nucleus under PCN challenge (Fig. 2h) while Dimethyl fumarate (DMF, Nrf2 activator and NF-κB inhibitor) displayed mild protection on PCN-stressed RAW cells (Fig. S2D).

### (+)JQ1 represses PCN-induced macrophage death by blocking RNS production

**Fig. 3:**
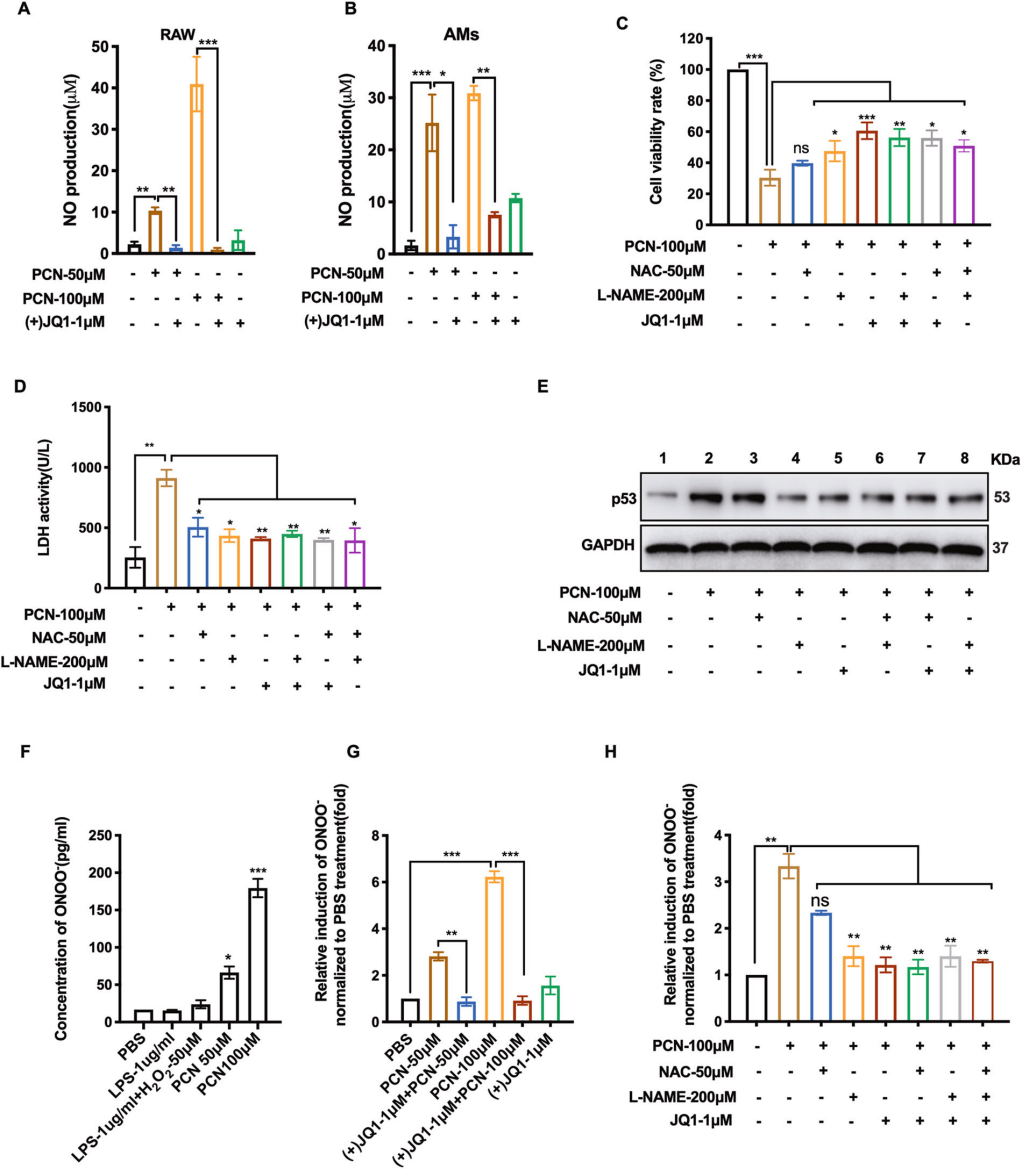
(+)JQ1 repressed PCN-induced RNS production. NO production was measured in the medium of **a** RAW cells and **b** AMs at 8 h PCN-post challenge. **c** CCK8 assay showing the cell viability of RAW cells with the independent or combined treatments of indicated reagents for 24 h. **d** LDH release and **e** p53 expression of RAW cells at 8 h post-treatment of independent or combined use of indicated reagents. **f** Concentration of ONOO^−^ measured in RAW cell medium at 8 h post-treatment of the indicated reagents. **g**, **h** Relative induction of ONOO^−^ in RAW cells at 8 h post-treatment of the indicated reagents with or without pre-treatment of (+)JQ1. (Data shown were the mean ± SD from three independent experiments. *P < 0.05, **P < 0.01, ***P < 0.001compared with indicated group. ns non-significant. One-way ANOVA test followed by *t*-test).

Although (+)JQ1 displayed effective inhibition on ROS production in PCN-challenged macrophage, fairly high dosages of NAC presented less resumed cell viability than (+)JQ1 did (Fig. 2b), implying the alternative factors might get involved. NO has been found to take part in macrophage-related immune response that is induced by LPS or pro-inflammatory cytokines^29^. Although the fluctuation of NO during PA infection is in debate^30,31^, previous study showed increased nitrite and iNOS expression in PA-infected human nasal tissues^32^. This is consistent with the substantial NO induction detected in PCN-challenged RAW cells and AMs, which is robustly inhibited by (+)JQ1 (Fig. 3a, b). We then applied NO synthases inhibitor L-NG-Nitroarginine methyl ester (L-NAME) to find L-NAME treatment alone could also rescue PCN-mediated cell death and damage (Fig. 3c, d, Fig. S3A). Moreover, while NAC alone could not totally eliminate PCN-induced p53 expression, L-NAME or (+)JQ1 alone inhibited p53 expression as effectively as the combinatory administration did (Fig. 3e), indicating the protection of (+)JQ1 might at least partially work through blocking PCN-mediated NO production (Fig. S3B). Given that the enzyme-free reaction between NO and superoxide anion (ROS) generates a much more cytotoxic RNS-ONOO^−^^33^, correlations between ONOO^−^ level and various stress stimulations were investigated in RAW cell culture where ONOO^−^ was found to be specifically induced by PCN (Fig. 3f). Consistent with NO change, (+)JQ1 diminished PCN-mediated ONOO^−^ production (Fig. 3g), where only L-NAME alone displayed comparable ONOO^−^ inhibition as (+)JQ1 did (Fig. 3h). Therefore, we concluded that (+)JQ1 rendered its protective effects on PCN-challenged macrophage via inhibiting ONOO^−^ and NO production.

### iNOS is required for PCN-mediated RNS production and macrophage death

**Fig. 4:**
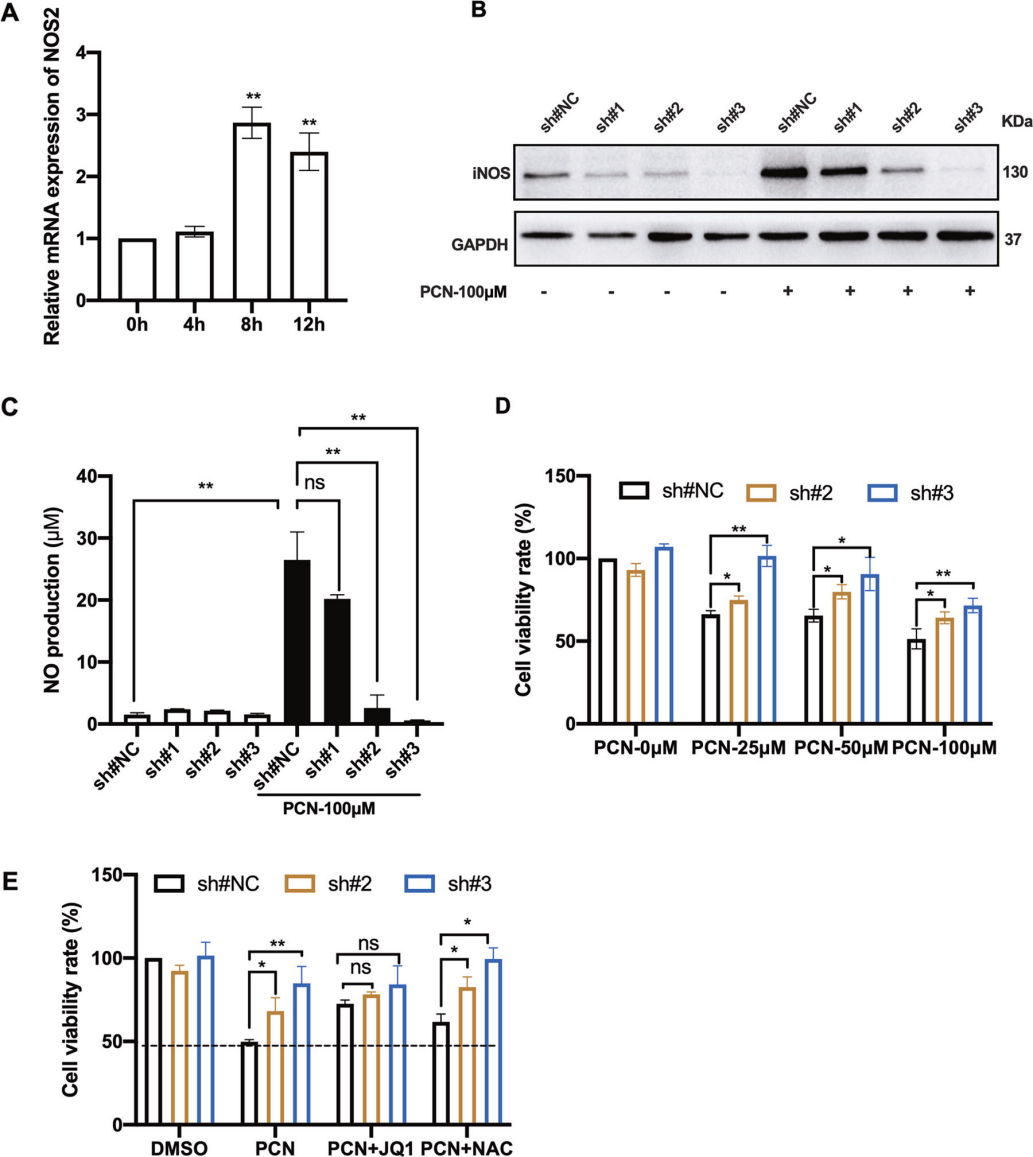
shRNA-mediated NOS2 silencing reduced PCN-mediated RNS production and macrophage death. **a** RT-qPCR showing the relative mRNA levels of NOS2 in RAW cells at indicated time points post-PCN treatment (100 μM). **b** Western blotting showing iNOS expression in RAW cells transfected with different shRNA with or without PCN treatment for 8 h. A non-target scrambled oligonucleotide sequence functioned as the negative control (sh#NC). **c** NO production measured in cell medium under the same condition in **b**. **d**, **e** Cell viability of NOS2 knock-down RAW cells treated with PCN or combination of indicated reagents for 24 h. (Data shown were the mean ± SD from three independent experiments. *P < 0.05, **P < 0.01, compared with indicated group, ns non-significant. One-way ANOVA test followed by *t*-test).

In-line with the dosage-dependent NO induction upon PCN stimulation (Fig. S3C), mRNA levels of *NOS2* were robustly up-regulated from 8 to 12 h post-PCN treatment (Fig. 4a). However, it was intriguing that co-incubation of RAW cells with PCN and LPS showed repressed NO production in the cell culture as previously reported^34^ (Fig. S3D). Lentivirus-mediated shRNA for *NOS2* knock-down was then conducted, showing effective silencing on iNOS expression even though it was largely induced by PCN treatment at the first place (Fig. 4b). Consistently, PCN-mediated NO production was inhibited by two of these shRNA sequences (sh#2 and #3) which showed more robust iNOS repression (Fig. 4c). Moreover, the rescued cell survival by these two shRNA sequences matched the extends of iNOS expression (Fig. 4d) which could not be enhanced by (+)JQ1 (Fig. 4e) verifying the involvement of iNOS in (+)JQ1-dependent macrophage survival protection upon PCN stress.

### (+)JQ1 restrains iNOS expression and Brd4 recruitment to *NOS2* promoter in PCN-stressed RAW macrophages

**Fig. 5:**
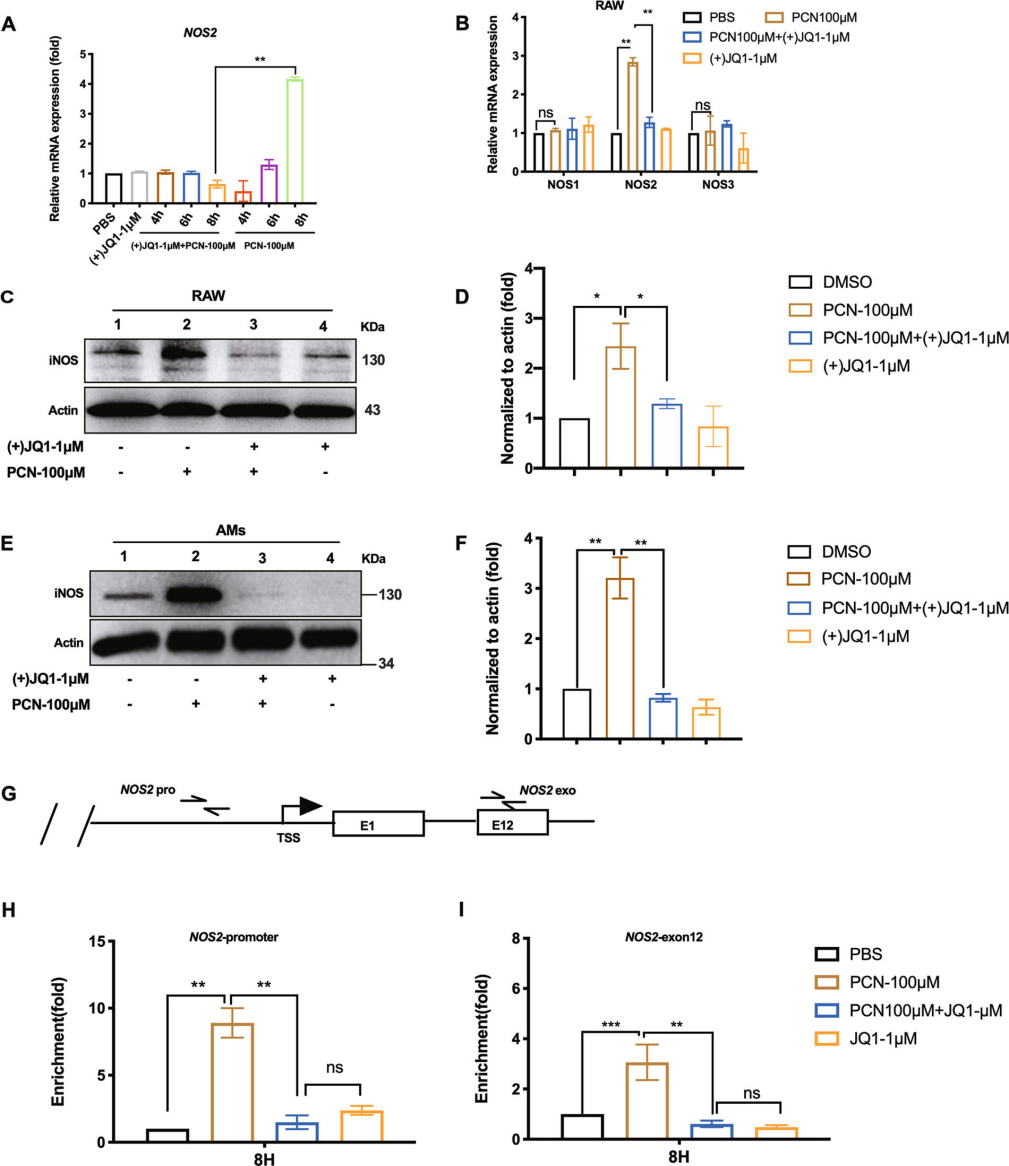
(+)JQ1 restrained iNOS expression and recruitment of Brd4 to NOS2 locus. **a** RT-qPCR showing the mRNA levels of NOS2 in RAW cells at indicated time points post-PCN challenge. **b** mRNA levels of NOS1/2/3 in RAW cells at 8 h post-PCN challenge. iNOS expression in RAW cells (**c**) or AMs (**e**) at 8 h post-PCN challenge. **d**, **f** Densitometry analysis of iNOS expression in **c**, **e**. **g** Illustration of ChIP-qPCR amplicons at NOS2 locus in RAW cells under the same condition as **b** (label “pro” as promoter region and label “exo” as the 12th exon region (E12); TSS: Transcription Star Site) using anti-Brd4 antibody. **h** qPCR showing the enrichment of Brd4 at NOS2 promoter and **i** the 12th exonic regions as illustrated in **g**. (Data shown were the mean ± SD from three independent experiments. *P < 0.05, **P < 0.01, compared with indicated group, ns, non-significant. One-way ANOVA test followed by *t*-test).

To date, three NOSes are found in mammalian to regulate NO production. iNOS has been shown to link with various cellular responses to outer stimulations as it is inducible to LPS and certain cytokines^13^. We showed (+)JQ1 effectively dampened PCN-mediated *NOS2* mRNA induction (Fig. 5a) at 8 h post-PCN challenge while two other NOS encoding genes, *NOS1* and *NOS3*, did not respond to PCN or (+)JQ1 treatment (Fig. 5b). In addition to mRNA level changes, Western blotting analysis agreed with RT-qPCR data in PCN-stressed RAW cells and AMs except the unexpected increase of iNOS from (−)JQ1 treated RAW cells (Fig. 5c–f). Brd4 has been found as the primary target of (+)JQ1 at *NOS2* promoter region during bacterial infection^23^. Our chromatin immunoprecipitation assay (ChIp) also showed significant enrichment of Brd4 at *NOS2* promoter and exon regions following PCN stimulation, which was completely canceled by (+)JQ1 (Fig. 5g–i). Thus, our data suggest (+)JQ1 repressed PCN-mediated iNOS expression and de-targeted Brd4 from *NOS2* loci.

### *BRD4* knock-down increases macrophage survival through interrupted RNS production

**Fig. 6:**
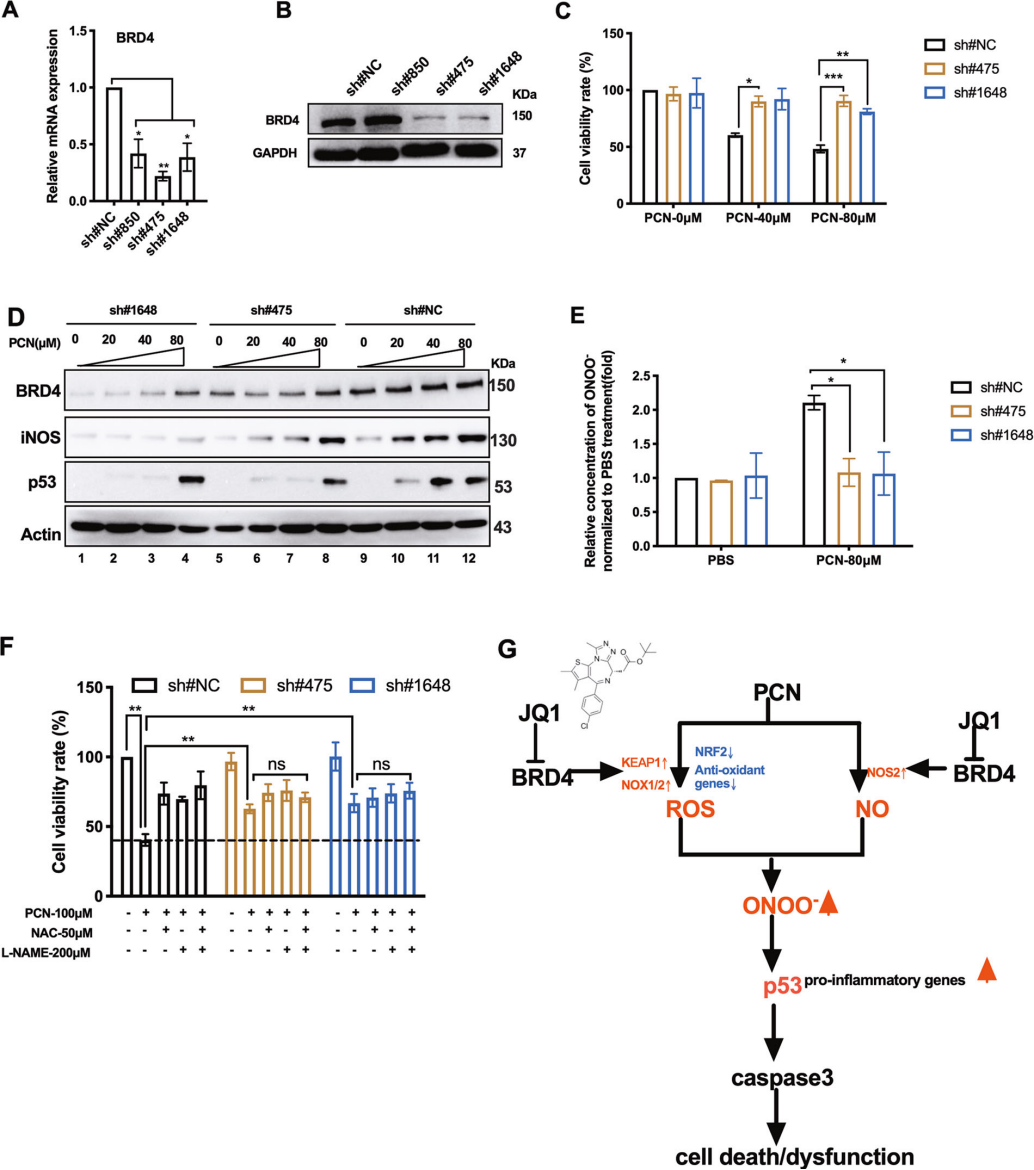
BRD4 knock-down increased macrophage survival and interrupted RNS production upon PCN stress. **a** The mRNA and **b** protein levels of Brd4 in RAW cells stably expressing individual shRNA sequences. **c** Cell viability of BRD4 knock-down cells (sh#475 and sh#1648) at 24 h post-treatment of PCN. **d** Western blot showing levels of Brd4, iNOS and p53 in BRD4 knock-down cells at 8 h post-treatment of PCN. **e** The relative production of ONOO^-^ induced in BRD4 knock-down cells at 24 h post-treatment of PCN. **f** Cell viability of BRD4 knock-down cells treated with independent or combined use of indicated reagents. A non-target scrambled oligonucleotide sequence functioned as the negative control (sh#NC). **g** The schematic diagram depicting the proposed mechanisms by which (+)JQ1 protects PCN-mediated macrophage cell death (see Discussion). (Data shown were the mean ± SD from three independent experiments. *P < 0.05, **P < 0.01, compared with indicated group. ns non-significant. One-way ANOVA test followed by *t*-test).

To further demonstrate that (+)JQ1 interrogates PCN cytotoxicity via inhibiting Brd4-mediated RNS production, we stably knocked down *BRD4* by lentivirus-mediated shRNA transduction. RT-qPCR analysis showed more than two folds reduction of *BRD4* mRNA by all three designed shRNA sequences (Fig. 6a) and two of them (sh#475 and sh#1648) displayed significant decrease on Brd4 protein levels (Fig. 6b). Knock-down of *BRD4* significantly increased cell survival under PCN challenge (Fig. 6c). Western blotting analysis showed elevated protein levels of Brd4, iNOS and p53 following increased doses of PCN were dramatically blocked by shRNA transduction (Fig. 6d). In-line with these changes, *BRD4* knock-down decreased PCN-induced ONOO^−^ production (Fig. 6e). Additionally, NAC, L-NAME or co-administration of them could not further enhance survival of *BRD4*-silenced RAW cells (Fig. 6f). Therefore, *BRD4* knock-down phenocopied (+)JQ1 treatment in rescuing PCN-induced macrophage death by interrupting RNS pathways. To this end, we demonstrated PCN cytotoxicity was carried out by increased intracellular ONOO^−^ accumulation through elevated ROS and NO. PCN directly generated ROS and disturbed cellular oxidant/anti-oxidant balance while elevated NO was mediated by induced iNOS. (+)JQ1, by targeting BET family member Brd4, inhibited PCN-dependent expression of *KEAP1*, *NOX1/2* and *NOS2* to restrain ROS and RNS, and subsequently down-regulated p53-dependent macrophage apoptosis (Fig. 6G).

### (+)JQ1 treatment orchestrates macrophage activities and attenuates PA-mediated lung infection

**Fig. 7:**
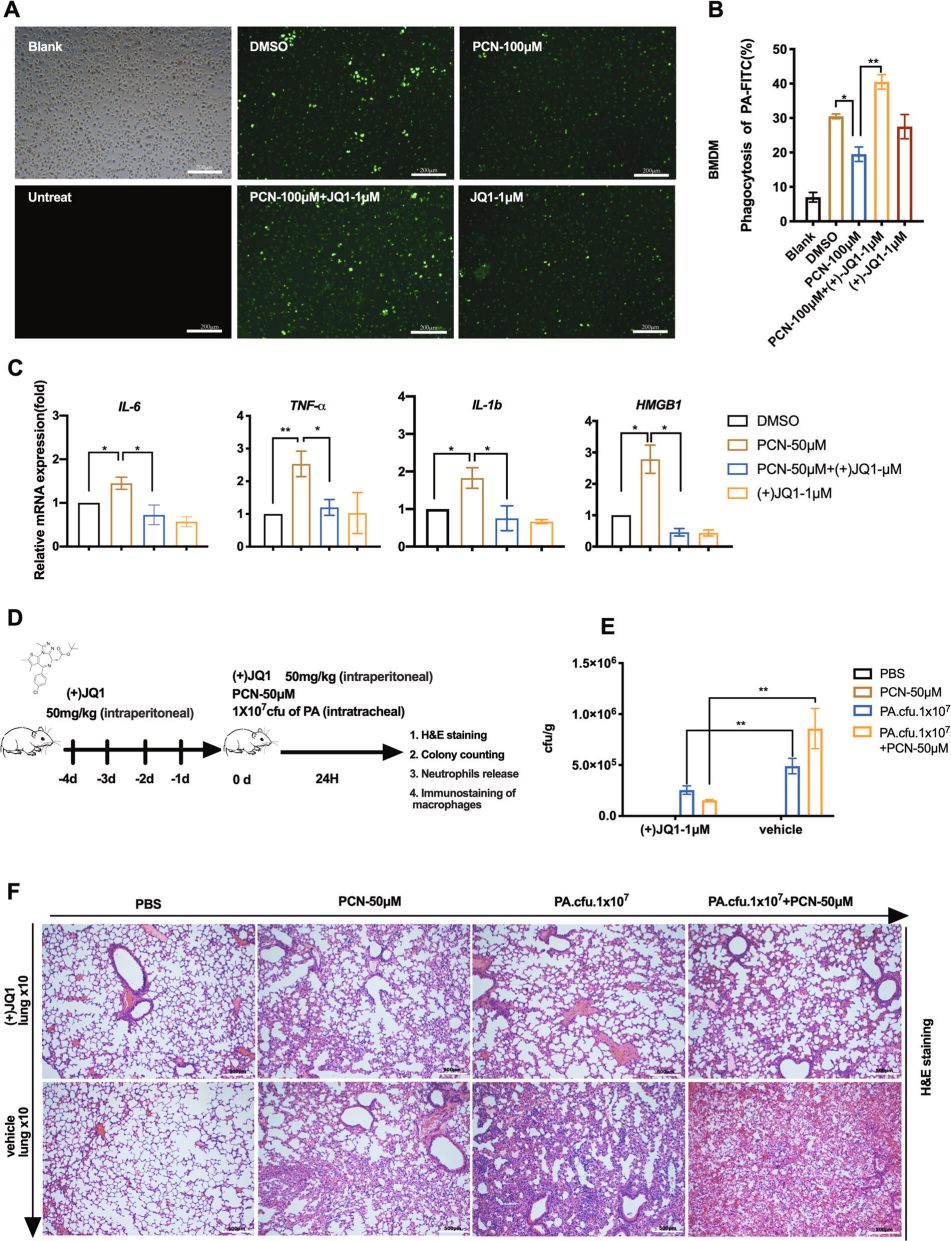
Effects of (+)JQ1 on macrophage activities and PA-mediated mice lung infection. **a** Fluorescence microscopy showing phagocytosis of FITC-labeled heat-inactivated PA (MOI 300:1) in PCN-stressed BMDMs with or without pre-treatment of (+)JQ1. **b** Flow cytometry quantification of engulfed FITC fluorescence in BMDMs shown in **a**. **c** RT-qPCR showing the relative expression of pro-inflammation genes in BMDMs at 12 h post-PCN challenge with or without pre-treatment of (+)JQ1. **d** The schematic diagram showing the experiment strategy of animal experiments: C57BL/6 mice were consecutively given intra-peritoneal injection of 50 mg/kg (+)JQ1 or 5% DMSO as control for 5 days (−4d to 0d). The intra-tracheal injection of 1.0 × 107 cfu PA with or without 50 μM PCN were given at 0d. **e** Bacteria counts in lung homogenates (Data were shown as means ± SD of cfu per gram of lung tissue from five mice in each group). **f** H&E staining showing histology change of infected lung (×10). (Data shown were the mean ± SD from three independent experiments. *P < 0.05, **P < 0.01, compared with indicated group, One-way ANOVA test followed by *t*-test).

We further investigated whether (+)JQ1 regulated macrophage activities upon PCN stress. FITC-labeled and heat-inactivated PA initiated phagocytosis with the increase of multiplicity of infection (MOI) (Fig. S4A). Fluorescence microscopy showed dramatic reduction of bacteria engulfment in both PCN-stressed bone marrow-derived macrophages (BMDMs) and RAW cells while pre-treatment of (+)JQ1 reversed such decrement (Fig. 7a and Fig. S4B). Flow cytometry analysis of FITC positive cells confirmed above observation in both cells (Fig. 7b and Fig. S4C). Additionally, previous studies have revealed (+)JQ1 repressed lung inflammation by inhibiting pro-inflammatory gene expression under multiple stress conditions^35,36^. Our RT-qPCR analysis also showed (+)JQ1 effectively suppressed PCN-induced pro-inflammatory gene expression in BMDMs (Fig. 7C). To exam the in vivo effects of (+)JQ1 in counteracting PCN-mediated PA infection in mice lung, we conducted intra-tracheal injection of PA with or without PCN following consecutive administration of (+)JQ1 (Fig. 7d). (+)JQ1 treatment effectively preserved macrophage population (CD68+) (Fig. S5A) while reduced bacteria burden and BAL fluid NO production from stressed lungs (Fig. 7e and Fig. S4D, E) without exerting possible antibiotics activity (data not shown), indicating roles of (+)JQ1 in elevating host immune defense. Moreover, H&E staining revealed that PCN alone enhanced PA-mediated lung inflammation (Fig. 7F vehicle group) which agreed with published data^37,38^. Pre-treatment of (+)JQ1 largely ameliorated such histological alternation in PCN, PA and co-stimulation groups respectively (Fig. 7f). Furthermore, the release of neutrophil from bone marrow has been considered the critical sign of inflammation as the infectious stimuli mobilized neutrophils from bone marrow reservoir^39^. We checked the proportion of neutrophils (Gr-1^+^/CD-11b^+^) in mice bone marrow by flow cytometry and found (+)JQ1 treatment effectively preserved neutrophil population after stimulation of PCN and/or PA respectively (Fig. S5B, C). In summary, our overall data revealed that (+)JQ1 fine-tuned macrophage functions and attenuated PA infection in vivo.

## Discussion

In this study, we demonstrated PCN induced macrophage stress via up-regulated intracellular ROS and NO pathways which were followed by elevated cytotoxic ONOO^−^. By inhibiting Brd4-mediated transcriptional activation of critical redox-oxidative genes (such as *KEAP1*, *NOX1/2*, and *NOS2*), BET protein inhibitor JQ1 protected macrophage survival and innate immune activities (Fig. 6g). PCN has been well known to induce oxidative stress in host cells by direct oxidizing NADPH and producing ROS^3,40^, which is testified in multiple cell types including neutrophil^2^ and endothelial cell^25^. However, the sufficiency of ROS in carrying out PCN cytotoxicity in macrophages is questionable, as iNOS specific inhibitor L-NAME and shRNA-mediated *NOS2* silencing was found to contribute in protecting macrophages (Figs. 3 and 4). We herein at the first time demonstrated iNOS-dependent NO was required for PCN-mediated macrophage death. In fact, although induced NO in monocytes servers as a bactericidal reagent as well as messenger molecule mediating immune response^41^, it has been reported that prompt fluctuation of NO could be highly damaging via p53-mediated apoptosis^33,42^. In spite of the dosage-dependent NO production following PCN stress (Fig. S3C), this toxin was also reported to inhibit LPS-induced NO^34^, which was reproducible in our system (Fig. S3D). More interestingly, compared with other extracellular stressors, we only observed PCN-specific stimulation of ONOO^−^ (Fig. 3F), making the underlying mechanism fairly intriguing for further investigation. Based on these findings, we proposed the bi-directional regulation of JQ1 in harnessing excessive NO and ROS production upon PCN stress to eliminate cytotoxic ONOO^−^. Given the fact that there still lacks evidence for anti-oxidant reagents (including NAC and Nrf2 activators) or NO synthase inhibitors (such as L-NAME) in clinical application due to their high EC50 (~mΜ) and side-effects^11,43^. Our results suggest that BET inhibitors (such as JQ1) which are currently in active clinical trials for cancer treatments with nM range of EC50^44^ have potential application in PCN detoxification, thereby supporting anti-PA infection treatment^45^.

JQ1 is well known for targeting BET family members to harness the overly activated transcription of key genes involved in multiple cellular process, such as *MYC*-dependent oncogenesis^46^. Our *BRD4* knock-down assay demonstrated that Brd4 definitely participated in JQ1-mediated cellular protection against PCN via regulating iNOS expression (Figs. 5b and 6d). Brd4 has been shown to localize on acetylated histone lysine residues and facilitate the formation of “Super enhancer (SE)” featured by concentrated chromatin-bound co-activators (mediator complex etc.) and enhancer histone markers (H3K4me1, H3K27ac)^47^. Forming SEs at specialized gene locus is essential for the rapid elevation of according cellular processes such as pro-inflammatory responses induced by TNF-α and LPS^48^. The ChIp assay showed that Brd4 was recruited at *NOS2* promoter where JQ1 could effectively de-targeted Brd4 occupancy (Fig. 5g–i). Besides iNOS, Keap1 is known to negatively control Nrf2-related anti-oxidant response while *NOX1/2* encode important enzymes for ROS generation, all of which were shown to be transcriptionally regulated by JQ1 under PCN stress (Fig. 2c). Based on these findings, we postulated that as the major virulence factor in PA infection, PCN may induce cellular stress via formation of SEs particularly in above stress-response genes. Besides BET proteins, other transcription regulators such as NF-κB and Nrf2 may get involved in mediating such process. However, the occupancy of related transcription factors in these loci is still needed to be further investigated. Noteworthily, JQ1 treatment largely enhanced expression of *NRF2* and its downstream anti-oxidant genes (Fig. 2c), which indeed agreed with previous studies showing JQ1 or silencing BET family members promoted Nrf2 expression via inhibiting Keap^16^ (Fig. 2d–g) as well as canceling the repressor function of BET proteins in Nrf2-containing complex^49^. Thus, the overall findings suggest JQ1 delicately exerts dual roles in manipulating oxidants and anti-oxidant gene transcription.

Besides rescuing PCN-induced cell death, we also observed JQ1 treatment orchestrated macrophage activities and attenuated PA-mediated lung infection. Macrophage is well known for its roles in defending against pathogen infection by direct engulfing invading organisms and recruiting other immune cells such as neutrophils^6^. Our in vitro assays demonstrated abolished PCN cytotoxicity in macrophages by JQ1 administration (Fig. 1) and Brd4 inhibition (Fig. 6c), which was consistent with the resumed macrophage survival in PCN/PA-stimulated mice lungs (Fig. S5A). Additionally, in vitro bacteria clearance data also agreed with the observation that JQ1 treatment preserved bacteria phagocytosis in both PCN-challenged BMDMs (Fig. 7a) and RAW cells (Fig. S4B). Although such phenomenon was not seen in lavaged alveolar macrophages (data not shown) and CD68 staining alone could not tell resident alveolar macrophages from its bone marrow-originated counterpart, it is still valid to hypothesis that during PA infection, the migrated macrophages from circulating monocytes were targeted by JQ1 for carrying out innate immune defense against PA. Moreover, previous studies have shown that JQ1 directly repressed pro-inflammatory gene expression in various stress conditions^35,49^. We observed PCN alone induced moderate lung inflammation featured by destructed alveoli as well as infiltrated granulocytes. Meanwhile, PA-mediated lung inflammation could be significantly facilitated by PCN (Fig. 7f). With JQ1 administration, the inflammation in all above conditions were effectively attenuated, which agreed with the pro-inflammatory gene expression changes observed in BMDMs (Fig. 7c). Noteworthily, besides generating inducible NO in stress-responded macrophages for bacteria killing, up-regulated *NOS2* expression is also consider the hallmark of pro-inflammatory activation (M1) of macrophages. To this end, the overly activated iNOS pathway may directly initiate tissue inflammation as well as cause RNS-mediated macrophage damage, both of which could be epigenetically repressed by Brd4 inhibition. Thus, our results uncovered that BET inhibitor JQ1 ameliorates PCN-mediated PA lung infection by fine-tuning macrophage activities via maintaining oxidative free radical homeostasis in vitro and in vivo, indicating a potential innate immune-supporting strategy against PA-related infection.

## Materials and methods

### Chemical regents and antibodies

PCN (R9532), N-Acetyl-L-cysteine (NAC, A7250), N^G^-Nitro arginine methyl　ester hydrochloride (L-NAME, N5751) and 2-hydroxy-propyl-β-cyclodextrin (128446-35-5), macrophage colony–stimulating factor M-CSF (M9170) were purchased from Sigma-Aldrich (St. Louis, USA). (+)JQ1 (HY-78695) and (−)JQ1 (HY-13030A), DMF (HY-17363), AG-1478 (HY-13524) were obtained from MedChem Express (Shanghai, China). Anti-actin and anti-Brd4 antibodies were provided by Abcam (Cambridge, UK). Antibodies for Nrf2(#12721), Keap1 (8047), iNOS (13120), Caspase 3 (9662), cleaved-caspase 3 (9661), p53 (2524) were provided by Cell Signaling Technology (Danvers, USA). Anti-CD68 antibody [FA-11](Alexa Fluor® 647) (201845) was provided by Abcam (Cambridge, UK). HOOK^TM^ Dye Labeling Kit (FITC) kit (86-080) was purchased from Sangon Biotech (Shanghai, China). Anexin V-FITC/PI cell apoptosis detection kit (FA101-01) was provided by TRANGEN BIOTECH (Beijing, China). PE-CyTM7-anti-CD11b (557743) and FITC-anti-Mouse Gr-1(553127) were provided by BD Phaningen^TM^. Simple ChIP^®^ Enzymatic Chromatin IP Kit (Magnetic Beads) (9003) was purchased from Cell Signaling Technology (Danvers, USA).

### Cell lines

RAW 264.7 cells were purchased from the Cell Bank of the Chinese academic of sciences (Shanghai, China) and grown in Dulbecco’s Modified Eagle Medium (DMEM) high glucose (4.5 g/L) and normal glucose (1 g/L) with 10% (v/v) fetal calf serum (Biological Industries) and 1% penicillin/streptomycin at 37 °C in humidified air with 5% CO_2_. Murine alveolar macrophage cells and murine bone marrow derived macrophage cells were cultured in PRM1640 with 10% (v/v) fetal calf serum (BBI) and 1% penicillin/streptomycin.

### Bacteria

*Pseudomonas aeruginosa* strain PAO1 is a gift from Sichuan Industrial Institute of Antibiotics (SIIA). After overnight growth in Luria–Bertani (LB) broth at 37 °C it was diluted by 50-fold with fresh medium and further incubated for 2 h. Bacteria were harvested by centrifugation at 3000 rpm for 10 min and then washed three times with phosphate buffered saline (PBS) for infection assay. Bacteria number was calculated by measuring the absorbance at 625 nm and absorbance of 0.6 AU represented 1 × 10^9^ cfu/ml.

### Experimental procedures

#### Isolation of murine alveolar macrophages and bone marrow derived macrophage

The murine alveolar macrophages and bone marrow cells were harvest as previously described^50^. In order to generate bone marrow derived macrophages, bone marrow cells were cultured in macrophage complete medium containing M-CSF (M9170, Sigma). After 7 days in culture, non-adherent cells are eliminated and adherent cells are harvested for assays.

#### Neutrophil percentage of bone marrow cells

Bone marrow cells were harvested and prepared to analysis by flow cytometry as previously described^51^. Briefly, cells were incubated with Rat anti-Mouse Gr-1 (FITC) and Rat anti-CD11b^+^ (PE-CyTM7) at 4 °C for 30 min. Mature neutrophils were gated as Gr-1^+^/CD11b^+^. Data were acquired using an C6 flow cytometry (BD Biosciences) and analyzed with the FlowJo software.

#### Animal experiment

Infection experiments were performed as described previously with modifications^23^. Briefly, 40 six-week-old male C57BL/6 mice were randomly assigned. The mice were given intraperitoneal injection of 50 mg/kg (+)JQ1 or 5% DMSO (diluted in 10% 2-hydroxy-propyl-β-cyclodextrin carrier) for 4 days prior to bacteria infection and the procedure continued throughout the duration of the experiment. The infection group were injected with 50 μl inoculum containing 50 μM PCN with or without 1 × 10^7^ cfu of viable PA and the vehicle controls were injected with 50 μl sterile PBS via the trachea according to the method described by Munder et al.^52^. Mice were anesthetized with a mixture of ketamine hydro-chloride (25 mg/kg body wt. Bayer Australia, Pymble, Australia) and Tiletamine-Zolazepam (60 mg/kg body wt. Virbac Australia, Peakhurst, Australia) via intraperitoneal injection. All mice were humanely euthanized 24 h after the infection. All in vivo studies were performed under an animal protocol approved by the university committee on use and animal protocol approved by Sichuan university. All animals received human care according to the criteria outlined in the “Guide for the Care and use of Laboratory Animals Chinese Version” (2006).

#### Quantitative bacteriology

After being anesthetized, the left lungs were dissected and homogenized in 5 ml of sterile PBS. The homogenate was then serially diluted by five times and cultured on LB plates. Colonies of PA were visualized after overnight incubation at 37 °C. Bacterial numbers of lung tissues were calculated as cfu per gram of lung tissue.

#### Histological studies

The right lungs were fixed in 4% PFA in PBS for 72 h and embedded in paraffin. One section was stained with hematoxylin and eosin (H&E) and examined in fifteen fields of views at a 10-fold magnification using IX73 microscopy (OLYMPUS). The other section was used for Immunofluorescence staining using anti-CD68 antibody [FA-11] (Alexa Fluor^®^647) and examined by LMS 710 confocal microscopy (Zeiss).

#### Collection of Broncho alveolar Lavage fluid

After being anesthetized, Broncho alveolar Lavage (BAL) fluid was collected by trachea in situ cannulation using a blunt-end 21-gauge needle. 1.0 ml of sterile PBS was instilled for three times and the fluid was collected by gentle aspiration. The total BAL fluid was centrifuged for 10 mins, and the supernatants were used for nitric oxide detection.

#### Phagocytosis assays

PA was heat-killed at 60 °C for 2 h. Bacteria were fluorescently labeled using FITC (HOOK^TM^) Dye Labeling Kit in the dark for 1 h on the shaking table. The labeled bacteria were washed twice in PBS to remove unbound label and re-suspended in PBS. Fluorescent-labeled bacteria were added to cells and incubated for 2 h.

Cells were seeded in confocal dish for at least 12 h and FITC-label PA (MOI 100:1 or MOI 300:1) were added to incubate for 2 h. Cells were washed with HBSS three times and fluorescence of extracellular particles was quenched by adding trypan blue (2% (volume/volume)) for 1 min. the sub-cellular localization of FITC-label bacteria was monitored under a LMS 710 confocal microscopy (Zeiss). The phagocytosis was investigated by IX73 microscopy (OLYMPUS) and quantified by C6 flow cytometry (BD Biosciences).

#### Quantitative real-time PCR (RT-qPCR)

Total RNA isolation was performed with TRIzol reagent (Invitrogen, Carlsbad, USA) and SYBR Green PCR reaction mixture (Bio-Rad, IQ, USA) was used according to the manufacturer’s instruction. Primers for individual genes are given in Table S1 in the supplemental material. Relative mRNA expression in different groups were normalized to *GAPDH* and calculated by 2^−ΔΔct^ method.

#### Chromatin immunoprecipitation assay

ChIP was performed as previously described^53^ by using a Simple ChIP^®^ Enzymatic Chromatin IP Kit. ChIP data were normalized to input and to the sample from untreated cells. Primers used for qPCR at indicated *NOS2* locus were referred to the previous publication^23^ and shown in Table S1.

#### Western blotting assay

Total protein was extracted by Protein Extraction Kit (KeyGen Biotech, Nanjing, China). The cytoplasmic and nuclear proteins were separated by Cytoplasmic and Nuclear Protein Extraction Kit (KeyGen Biotech, Nanjing, China). The protein concentration was determined by BCA Protein Assay kit (Beyotime, China). Western blotting analyses were performed with the primary antibodies as indicated and followed by horseradish peroxidase-conjugated secondary antibodies. Signals were detected by enhanced chemiluminescence (Merck Millipore, Bedford, MA, USA) and exposed using ChemiDoc^TM^ MP Imager (Bio-Rad, USA). The integral optical density of each sample was measured by Image J.

#### ROS and RNS measurement

The intracellular ROS level was determined by using DHE assay kit (Keygen Biotech, China) following the manufacturer’s protocol. Briefly, cells were washed twice with PBS and incubated with DHE (10 μM) at 37 °C for 20 min in a darkroom for image analysis by IX73 microscopy (OLYMBUS) and fluorescence quantification by C6 flow cytometry (BD Biosciences).

The NO production was measured using Griess Reagent Kit for Nitrite Determination (Beyotime, Nanjing, China) according to the manufacturer’s protocol. Briefly, the 50 μl cell lysate or culture supernatant was mixed with an equal volume of Griess reagent I and then reagent II at room temperature. The absorbance of mixture was immediately measured at 540 nm.

An Enzyme-linked Immunosorbent Assay Kit for ONOO^−^ (TW reagent, Shanghai) was used to measure the ONOO^−^ concentration in cell culture following the manufacturer’s instruction. Briefly, 50 μl centrifuged cell culture supernatant was taken and mixed with 100 μl of Enzyme conjugate to incubate for 60 minutes at 37 °C. After four times wash, 50 μl Substrate A and 50 μl Substrate B were added to each well. In all, 50 μl Stop Solution were added after 15 min gentle mix at 37 °C in dark. Optical Density (OD) at 450 nm was read by using microtiter plate reader (Thermo Scientific, USA) within 15 minutes and ONOO^−^ concentration was calculated according to the standard curve.

#### Lactate dehydrogenase release assay (LDH assay)

Cell culture supernatant was harvested to detect the cytoplasmic enzyme LDH using a Cytotoxicity Detection Kit PLUS (LDH Roche, USA) following the manufacturer’s instructions. The LDH activity was determined as (OD_A_ − OD_control_)/ (OD_standard_ − OD_blank_)× 0.2 μmol × 1000(U/L)

#### Cell viability and apoptosis measurement

Cell viabilities were determined using CCK8 dye (Colleagues Association, Japan). Briefly, 3 × 10^4^ cells were seeded in a 96-well flat-bottomed plate at 37 °C overnight. After treatment, 10 μl CCK8 dye was added to each well and incubated at 37 °C for 2 h. The absorbance was finally determined at 450 nm using microplate reader. The cell viability was calculated as (OD_A_ − OD_blank_)/ (OD_control_ − OD_blank_) × 100%.

Anexin V / PI stain kit was applied to detect apoptosis on RAW cells. Briefly, cells were collected by centrifugation and re-suspend in 500 μl binding buffer. In all, 5 µl of Anexin V-FITC and 5 µl of propidium iodide (PI) were added and incubated at room temperature for 15 min in the dark. Then C6 flow cytometry (BD Biosciences) was used for analyzing.

#### Establishment of cell lines expressing short hairpin RNA

short hairpin RNA (shRNA) sequences against *BRD4* or *NOS2* gene were designed and sub-cloned into lentivirus constructs (LV3 H1/GFP&Puro). Virus was produced by Gene Pharma Co., Ltd. (Shanghai, China). To generate stable knock-down cells, RAW cells were infected with 20 µl viral supernatant (1 × 10^8^ UT/ml) and 5 µg/ml polybrene (Han Heng Biotechnology Co., Ltd.) for 72 h followed by 2 µg/ml puromycin (Han Heng Biotechnology Co., Ltd.) selection. After 5 to 8 weeks the single colonies of puromycin-resistant RAW cells were harvested and characterized for gene silencing by RT-qPCR or Western blot. shRNA sequences used in the study were shown in Table S1.

#### Small interfering RNA transfection

p53-specific small interfering RNA (siRNA) was purchased from CST (SignalSilence®p53 siRNAII, USA). An unrelated siRNA (SignalSilence®Control siRNA #6568) was used as a control. Cell transfection was performed using jetPRIME^®^ transfection reagent for 72 h (Polyplus Transfection, USA) following the manufacturer’s instruction.

### Statistical analysis

Data are expressed as mean ± standard deviation of individual values from three independent experiments. The one-way ANOVA and two-tailed unpaired Student’s *t*-test was used to calculate the differences between two groups by Graphpad Prism 6. And *P* < 0.05 was considered as statistically significant.

## Supplementary information

Supplement Figure legends

Table S1

Figure S1

Figure S2

Figure S3

Figure S4

Figure S5
