# Supplementary material for: Brd4 inhibition ameliorates Pyocyanin-mediated macrophage dysfunction via transcriptional repression of reactive oxygen and nitrogen free radical pathways
Source: Cell Death Dis. 2020 Jun 15;11(6):459. doi: 10.1038/s41419-020-2672-0 (PMC7295752; doi:10.1038/s41419-020-2672-0)
Supplement: Supplementary file 1 — Supplement Figure legends [file 41419_2020_2672_MOESM1_ESM.docx]

**Supplementary Figure S1.** (A) Western blotting showing p53 and cleaved-caspase 3 expression in RAW cells transfected with indicated siRNA with or without PCN treatment for 8h. A non-target scrambled oligonucleotide sequence functioned as the negative control (si-control). (B) Cell viability of P53 knock-down cells at 24h post-treatment of PCN. (C) Flow cytometry analysis and (D) Quantified apoptosis rates of double-stained RAW cells at 8h post-PCN challenge. AnnexinV-FITC (apoptotic fraction: Q2 and Q3 quadrants) and PI (necrotic fraction: Q1 quadrant). (Data shown were the mean ± SD from 3 independent experiments. *P < 0.05, **P < 0.01 compared with indicated group, One-way ANOVA test followed by t-test).

**Supplementary Figure S2.** (**A**) Representative images showing DHE-stained intracellular ROS in AMs cells at 3h post-PCN challenge in the indicated groups. (**B**) Relative mRNA levels of *EGFR* in RAW cells at 8h post-PCN challenge. (**C** and **D**) Cell viability of PCN-challenged RAW cells with the pre-treatment (pre) or co-incubation (co) of AG-1478 and DMF respectively. (Data shown were the mean ± SD from 3 individual experiments. *P < 0.05, **P < 0.01, compared with indicated group. ns, non-significant. One-way ANOVA test followed by t-test).

**Supplementary Figure S3.** (**A**) Cell viability of RAW cells treated with independent or combined administration of indicated reagents for 24h. (**B, C** and **D**) NO production measured in medium with the independent or combined treatments of indicated reagents for 24h. (Data shown were the mean ± SD from 3 independent experiments. *P < 0.05, **P < 0.01, ***P<0.001 compared with indicated group. ns, non-significant. One-way ANOVA test followed by t-test).

**Supplementary Figure S4.** (**A**) Engulfment of FITC-labeled heat-inactive PA by RAW cells was monitored by confocal microscopy at 2h post-treatment. Peri-nuclear localization of engulfed bacteria were indicated by white arrows (magnification, ×63). (**B**) Fluorescence microscopy showing phagocytosis of FITC-labeled heat-inactivated PA (MOI 300:1) in PCN-stressed RAW cells with or without pre-treatment of (+)JQ1 (magnification, ×10). (**C**) Phagocytosis rate of RAW cells under indicated treatments was measured by Flow cytometry. (**D**) Bacteria colony formation shown from lung homogenates on LB plates (**E**) NO level in Broncho alveolar Lavage (BAL) fluid from lung-infection mice. (Data shown were the mean ± SD from 3 independent experiments. *P < 0.05, **P < 0.01, ***P<0.001 compared with indicated group, One-way ANOVA test followed by t-test).

**Supplementary Figure S5.** (**A**) Immuno-histochemical staining of lung macrophage populations (CD68+ red, DAPI blue, magnification, ×10) from mice treated as indicated in **Fig.7D**. (**B**) Flow cytometry showing neutrophil populations (Q2 quadrant: Gr-1^+^/CD11b^+^ cells) in bone marrow from the left leg of mice treated as indicated in **Fig.7D**. (**C**) Statistics of neutrophil percentage from (**A**) (Data shown were the mean ± SD from 3 independent experiments. *P<0.05 compared with indicated group, One-way ANOVA test followed by t-test).
