# Supplementary material for: Brd4 inhibition ameliorates Pyocyanin-mediated macrophage dysfunction via transcriptional repression of reactive oxygen and nitrogen free radical pathways
Source: Cell Death Dis. 2020 Jun 15;11(6):459. doi: 10.1038/s41419-020-2672-0 (PMC7295752; doi:10.1038/s41419-020-2672-0)
Supplement: Supplementary file 2 — Table S1 [file 41419_2020_2672_MOESM2_ESM.docx]

**Table S1. primer sequences used in the paper**

|  | **Gene** | **Forward 5'-3'** | **Reverse 5'-3'** |
| --- | --- | --- | --- |
| **RT-qPCR** | **NOX1** | GCTGGATTTGAGAGCGTTGC | GGTGGTATCTAGGGCTATGCT |
|  | **NOX2** | TGGCTCCACTGGGAATTGC | CAAACCCGGCATCATGGGA |
|  | **CATALASE** | TGTTGCTGGAGAATCGGGTTC | TCCCAGTTACCATCTTCTGTGTA |
|  | **Mn-SOD** | AAACGTGACTTTGGTTCCTT | TCCCAGTTACCA CTTCTGTGTA |
|  | **HO-1** | GCCCTGCCCTTCAGCAT | AGCTGCCACATTAGGGTGTCTT |
|  | **KEAP1** | TGCCCCTGTGGTCAAAGTG | GGTTCGGTTACCGTCCTGC |
|  | **NRF2** | TTCCTCTGCTGCCATTAGTCAGTC | GCTCTTCCATTTCCGAGTCACTG |
|  | **NOS1** | AGCTCCTGGAACGACTACCTG | CCGGCACACAGCTCTAGTG |
|  | **NOS2** | GTTCTCAGCCCAACAATACAAGA | GTGGACGGGTCGATGTCAC |
|  | **NOS3** | GGCTGGGTTTAGGGCTGTG | CTGAGGGTGTCGTAGGTGATG |
|  | **BRD4** | GTGGGAGGAAAGAAACAGGGACA | AGGAGGAGGATTCGGCTGAGG |
|  | **IL-6** | GGCGGATCGGATGTTGTGAT | GGACCCCAGACAATCGGTTG |
|  | **TNF-α** | CACAGAAAGCATGATCCGCGA | CGGCAGAGAGGAGGTTGACTTT |
|  | **HMGB1** | GCCAGGAGAGCACAAGACAA | GCAACGACACCAATGGATAAACC |
|  | **EGFR** | GCCATCTGGGCCAAAGATACC | GTCTTCGCATGAATAGGCCAAT |
|  | **IL-1β** | AACCTGCTGGTGTGTGACGTTC | CAGCACGAGGCTTTTTTGTTGT |
| **CHIP-PCR** | **NOS2 prox** | GTCCCAGTTTTGAAGTGACTACG | GTTGTGACCCTGGCAGCAG |
|  | **NOS2 exon** | CCACACAGCCTCAGAGTCCT | CAACATCTCCTGGTGGAACA |
| **shRNA** | **shBRD4#475** | GCTCAAGACACTATGGAAACA |  |
|  | **shBRD4#850** | GGTACCAAACACAACTCAAGC |  |
| **shRNA** | **shNOS2#1** | GGCTGTCACGGAGATCAATGT |  |
|  | **ShNOS2#2** | GCACAGAATGTTCCAGAATCC |  |
