# Supplementary figures and images for: Brd4 inhibition ameliorates Pyocyanin-mediated macrophage dysfunction via transcriptional repression of reactive oxygen and nitrogen free radical pathways
Source: Cell Death Dis. 2020 Jun 15;11(6):459. doi: 10.1038/s41419-020-2672-0 (PMC7295752; doi:10.1038/s41419-020-2672-0)

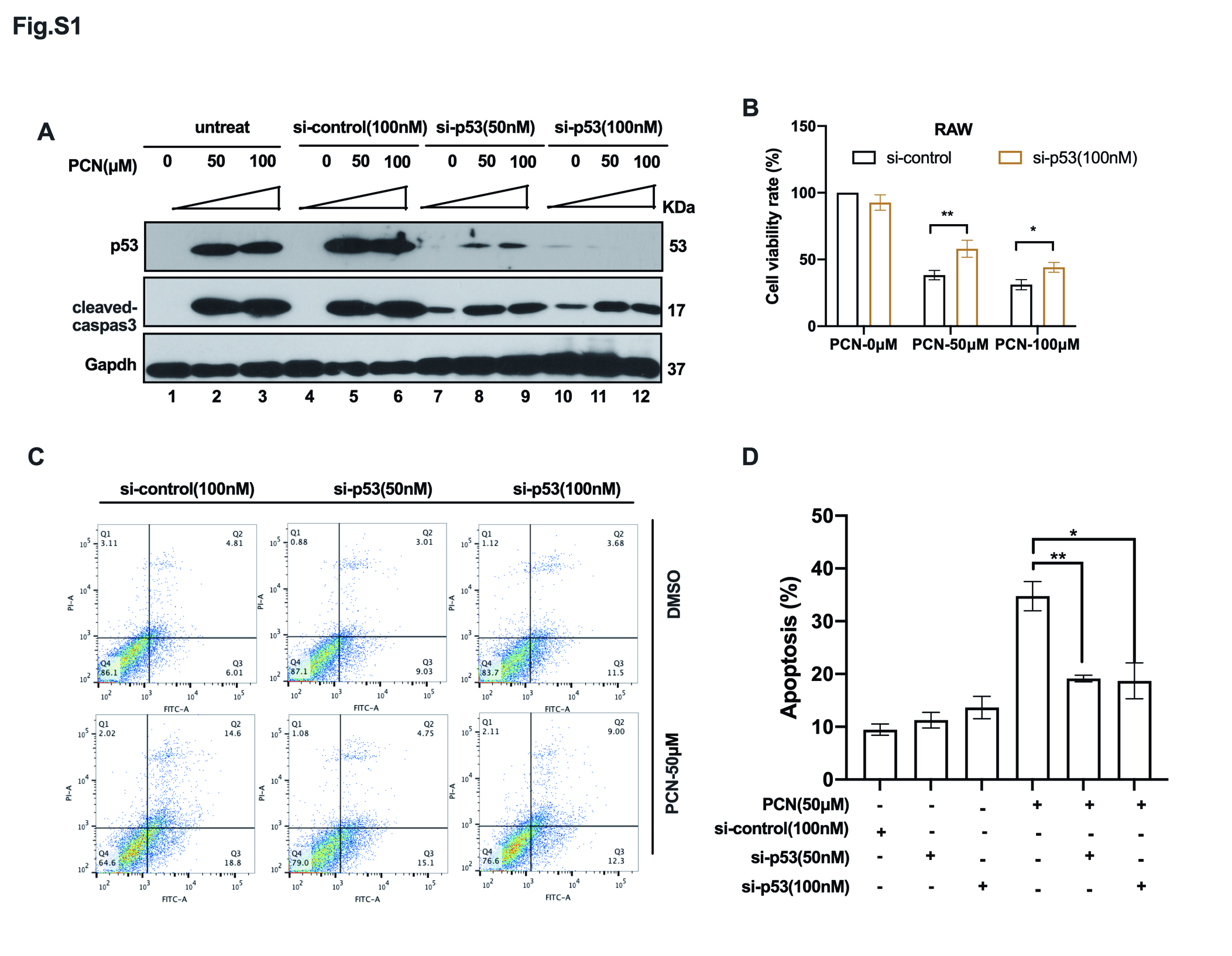

Supplement: Supplementary file 3 — Figure S1 [file 41419_2020_2672_MOESM3_ESM.tif]

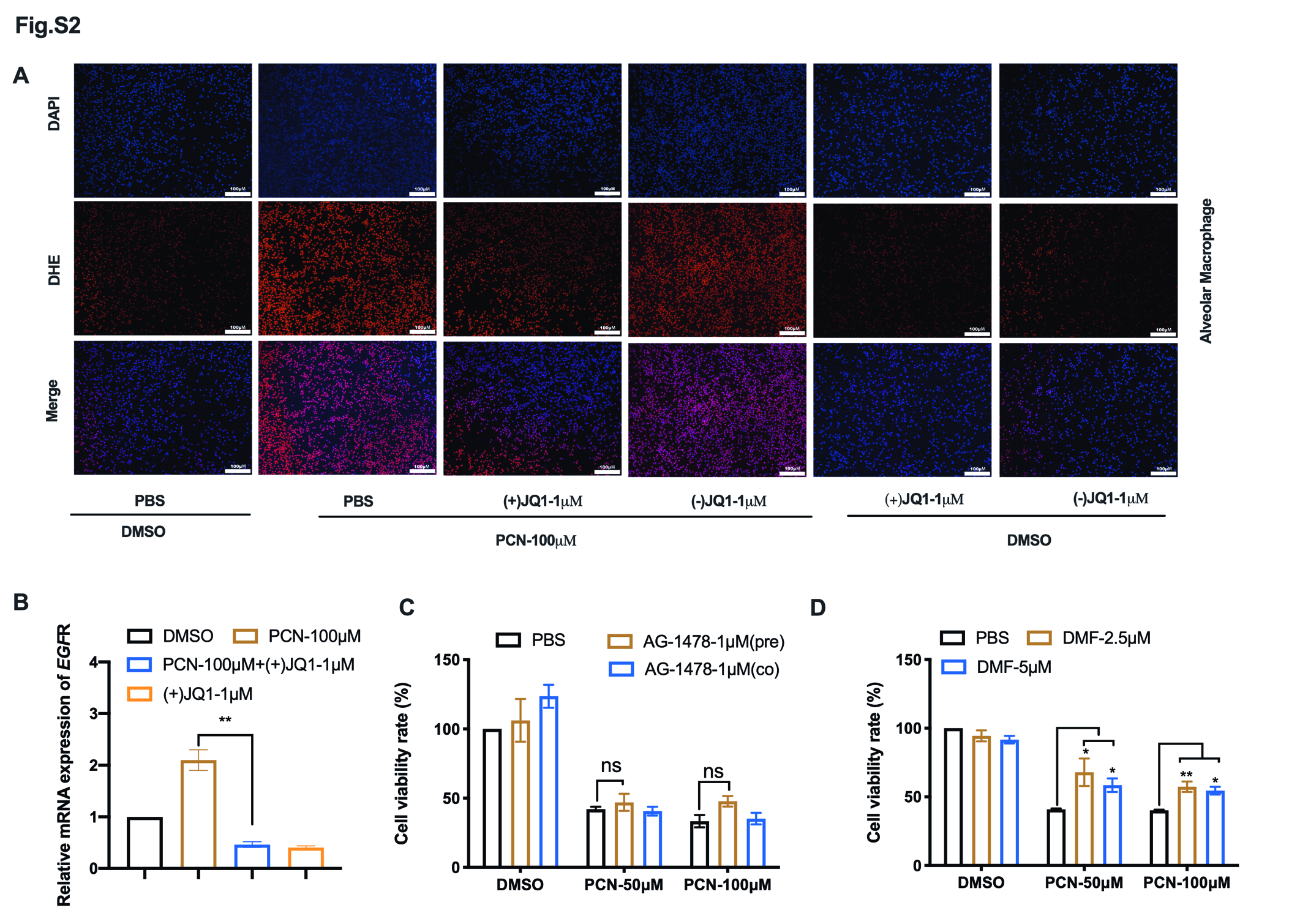

Supplement: Supplementary file 4 — Figure S2 [file 41419_2020_2672_MOESM4_ESM.tif]

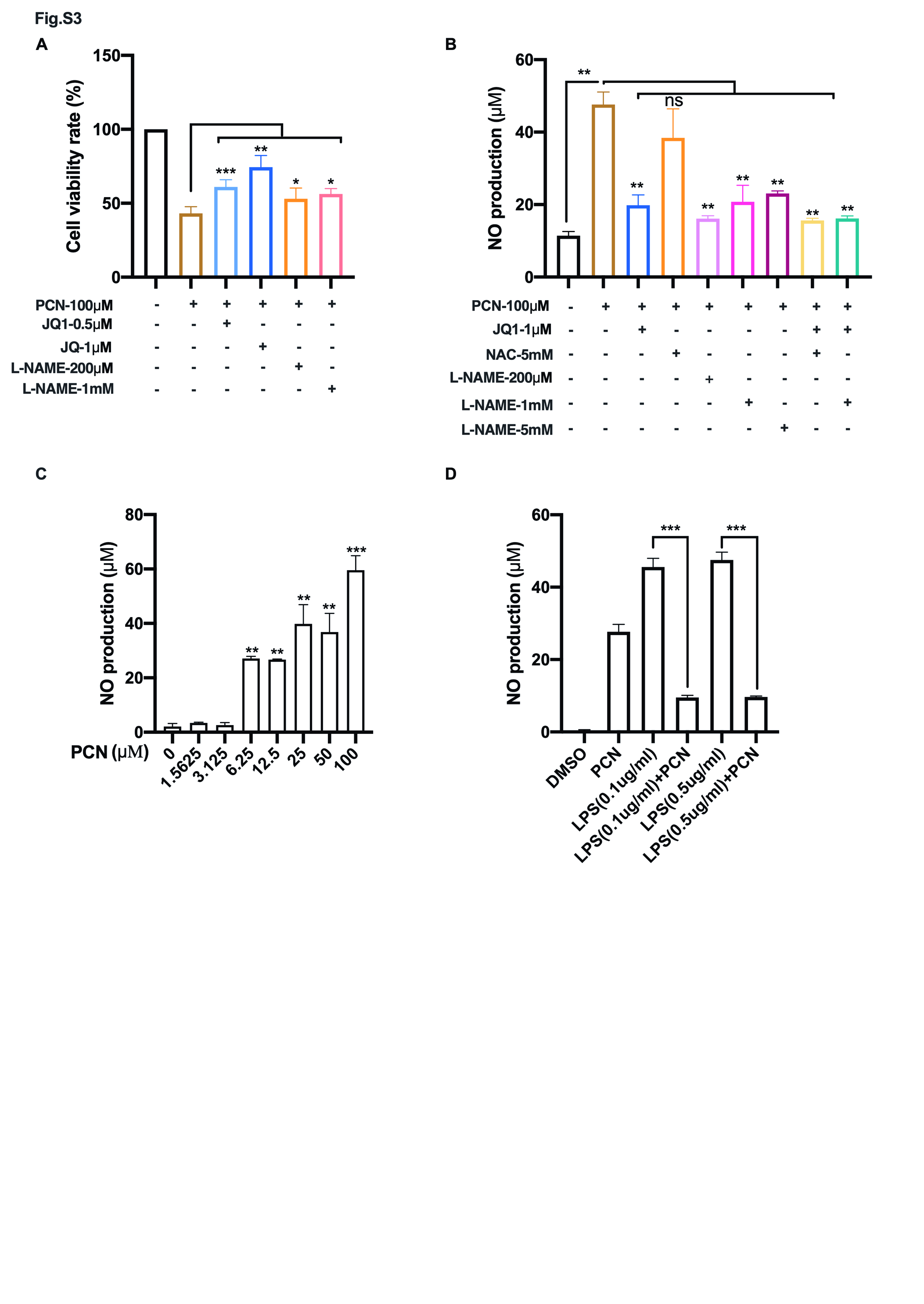

Supplement: Supplementary file 5 — Figure S3 [file 41419_2020_2672_MOESM5_ESM.tif]

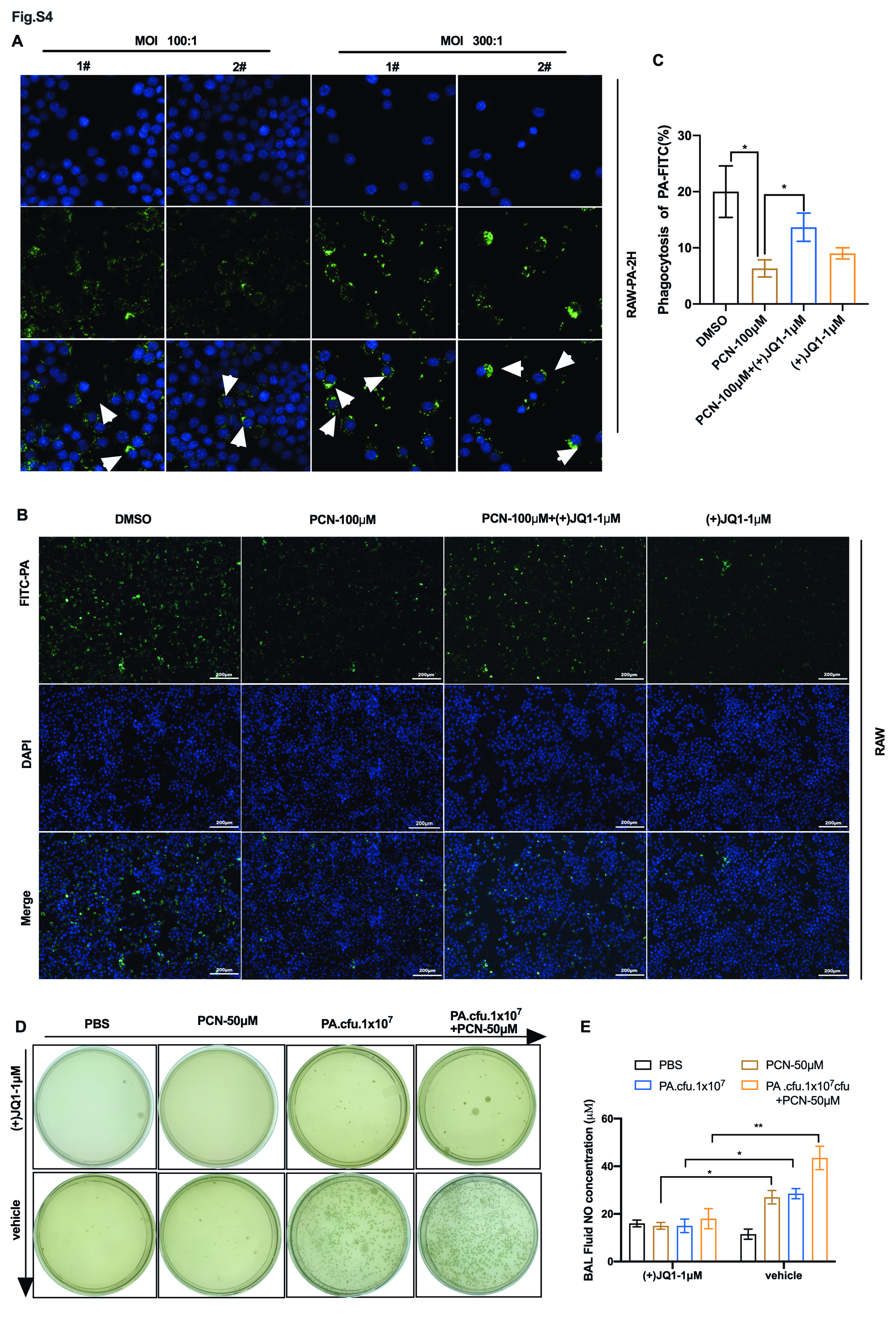

Supplement: Supplementary file 6 — Figure S4 [file 41419_2020_2672_MOESM6_ESM.tif]

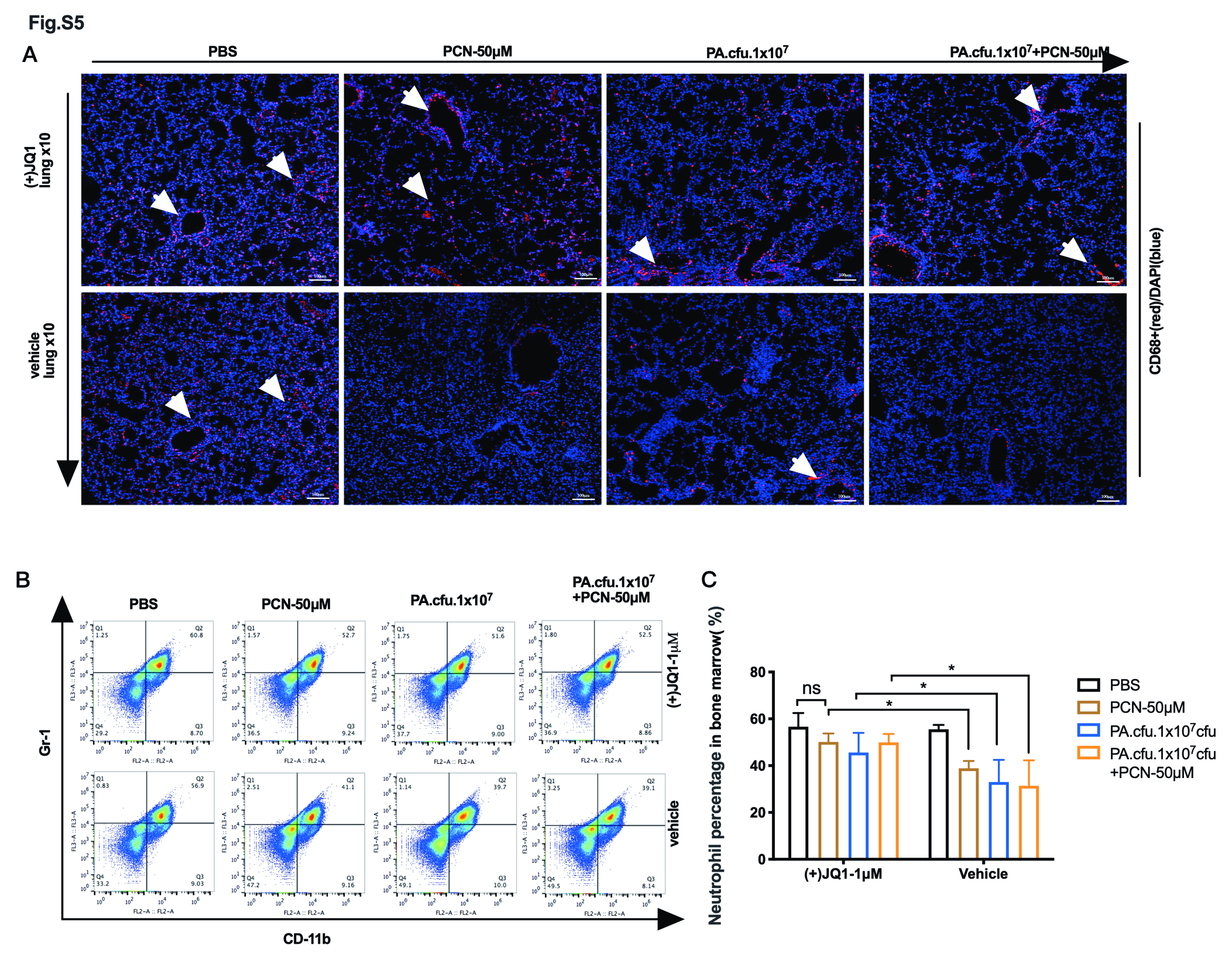

Supplement: Supplementary file 7 — Figure S5 [file 41419_2020_2672_MOESM7_ESM.tif]
